# Indicators of Absolute and Relative Changes in Skeletal Muscle Mass during Adulthood and Ageing

**DOI:** 10.3390/ijerph17165977

**Published:** 2020-08-18

**Authors:** Milivoj Dopsaj, Filip Kukić, Marina Đorđević-Nikić, Nenad Koropanovski, Dragan Radovanović, Dragan Miljuš, Dane Subošić, Milena Tomanić, Violeta Dopsaj

**Affiliations:** 1 Faculty of Sport and Physical Education University of Belgrade, 11030 Belgrade, Serbia; milivoj.dopsaj@fsfv.bg.ac.rs (M.D.); marina.nikic@fsfv.bg.ac.rs (M.Đ.-N.); 2 Institute of Sport, Tourism and Service, South Ural State University, 454080 Chelyabinsk, Russia; 3 Police Ports Education Center, Abu Dhabi Police, 253 Abu Dhabi, UAE; 4 Department of Criminalistics, University of Criminal Investigation and Police Studies, 11080 Belgrade, Serbia; nenad.koropanovski@kpu.edu.rs (N.K.); dane.subosic@kpu.edu.rs (D.S.); 5 Faculty of Sport and Physical Education University of Nis, 18000 Nis, Serbia; fiziologija@fsfv.ni.ac.rs; 6 Epidemiology and Surveillance Department, Institute of Public Health of Serbia “Dr Milan Jovanović Batut”, 11000 Belgrade, Serbia; dragan_miljus@batut.org.rs; 7 Institute of Hygiene and Medical Ecology, Medical Faculty University of Belgrade, 11000 Belgrade, Serbia; milena.tomanic@med.bg.ac.rs; 8 Department of Biochemistry, Faculty of Pharmacy University of Belgrade, 11221 Belgrade, Serbia; violeta.dopsaj@pharmacy.bg.ac.rs

**Keywords:** proteins, skeletal muscle loss, sarcopenia, physical activity, quality of life

## Abstract

This study aimed to explore the set of variables related to skeletal muscle mass (SMM) in both sexes, and to create age- and sex-related models of changes in SMM, using the most representative indicator of muscular status. Body composition was assessed in 8733 subjects (♀ = 3370 and ♂ = 5363), allocated into subsamples according to age: 18–29.9, 30–39.9, 40–49.9, 50–59.9, 60–69.9, and 70.0–79.9 years. Nine variables were used: protein mass, protein percent, protein mass index, SMM, percent of SMM, SMM index, fat-free mass, fat-free mass index, and protein/fat index. Univariate and multivariate analysis of variance (ANOVA and MANOVA) were used to determine between- and within-sex difference in all variables by age. Correlation analysis established the relationship between age and muscularity variables. Principal Component Analysis extracted the variables that loaded highest in explaining muscularity, while regression analysis determined the linearity of association between the age and indicators of muscular status. Variables SMMI and PSMM were extracted as the most sensitive to age, with SMMI being gender-independent while showing the parabolic and sinusoidal form of change as function of ageing in males and females, respectively; and PSMM being sex-dependent while showing a linear trend of decrease in both sexes.

## 1. Introduction

Stature and body weight are the most commonly used measurements in growth studies from childhood to early adulthood (i.e., birth to 19 years of age). Both dimensions can be systematically measured on regular bases in hospitals, schools or sports clubs with the purpose of following growth status through progress of body dimensions [1]. Longitudinal body dimensions (i.e., body height and length of the limbs) and body width (i.e., hip and shoulder width) follow a particular specific pattern during growth, while body dimensions such as body composition have specific patterns of changes during the life span [2]. Generally, body composition is sex-specific [3] and has been found to be negatively associated with aging [4,5,6], whereby lifestyle, nutrition, physical activity and exercise can influence significant positive as well as negative changes in body composition [7,8,9,10]. This can be analyzed on multiple levels (i.e., whole body, tissue, molecular, and atomic level), which depend on the availability of devices and methods that can often be costly [1,10,11,12]. The validity of these devices and methods further depends on the estimation formulas that they use, among which some are more and some less precise [13]. However, a few of them were validated against the gold standard procedure and were used for the development of cut-off values for certain components of body composition [14].

In the last two decades, significant technological progress developed techniques and equipment that are in use for defining the individual components of body composition, which provided the conditions and new possibilities for complex insight into body composition at a relatively low cost, which is fast and reliable [1,15,16]. According to the actual standards, air-displacement plethysmography (Bod Pod) and dual-energy X-ray absorptiometry (DEXA) were accepted as reference methods (gold standard) for the validity of body composition assessment and analysis [12,17,18,19]. In that regard, multi frequency bioelectric impedance analysis (BIA) method for assessment of body composition was found to be valid and reliable, and thus became widely used in clinics, public health, occupational health, and sports medicine [8,10,12,20,21]. Moreover it is considered a portable alternative to DEXA [14]. Therefore, the results obtained from age-related studies could be used for the development of fundamental values that serve as the basis for defining evidence-based scientific knowledge related to biological, physiological, functional and physical changes in the health of humans during aging and lifespan [2,4,6,21,22]. In addition, results from the studies on growth and ageing could be cross-validated between the cultures and socio-economic statuses, whereby reference values could be calculated with well-defined precision and error of measurement. This means that body composition measures could be defined relative to age, indicating healthy reference values as well as those that may lead to conditions such as malnutrition, sarcopenia or any kind of disability [19,23,24,25]. 

Aging is an irreversible, continuous, and biologically regulated gradual process characterized by great variability among individuals, different body systems and organs of the same individual [23]. The most important component of body composition associated with nutritional status, physical activity level and expression of muscle strength and power is skeletal muscle mass [26,27,28]. It has been previously found that low muscle strength is associated with mortality mostly due to low muscle mass that occurs during aging as either a biological or pathological process, which may lead to sarcopenia [4,29,30]. Sarcopenia is a characteristic of biological aging that involves a progressive loss of skeletal muscle mass (pre-sarcopenia), as the first stage, loss of muscular strength (sarcopenia), as the second stage, and decrease in physical performance (severe sarcopenia) as the final stage [14,23,24,31,32]. Moreover, the evidence to suggest that sarcopenia is in close relation with dynapenia, as a more constant factor in compromised wellbeing in old age [33]. In that regard, following the trends in skeletal muscle mass (SMM) or protein mass (PM) that occur during adulthood and aging are very important as they may provide enough evidence for setting the cut-off values for the normal muscular status of adult human of both sexes. Accordingly, the outliers with values either above or below the normal status may be the ones with well-developed or underdeveloped muscle mass (i.e., pre-pathologically and pathologically low muscle mass).

The assessment of various indicators of SMM may provide direct information about SMM status as well as valuable information about the tissue potential for the physical activity and metabolic processes of humans [8,20,28]. The possibility of having accurate information about the SMM status is important in diagnostics and prognostics of overall health, nutritional status and physical fitness as a potential for healthy aging, or the possible prevention of obesity. Note that obesity is among the leading risk factors for noncommunicable diseases such as hypertension, diabetes, coronary artery disease, and hyperlipidemia [18,26,34], whereby SMM is responsible for the metabolism that is needed for a reduction in body fatness. Therefore, better understanding of the variability and trends in body composition characteristics related to SMM and its constituents (i.e., PM), throughout adulthood and ageing is essentially important. A national data survey study, where national data could provide a valid scientific basis for specific, sensitive, age- and gender-dependent diagnostic purposes, could aim to increase the effects of health prevention and intervention. Moreover, the efficient development of a curative measure may have an impact on improvement in morphological profile in individuals through permanent systematic screening of muscularity (i.e., absolute and relative skeletal muscle content) status.

Although some effort has been made in the evaluation of SMM in different age categories [4,6], evidence and cross-cultural data are scarce, especially considering different indicators of muscularity. For example, people of different sizes may possess different absolute values of SMM only because of their body size (i.e., larger males compared to smaller males or males compared to females). Furthermore, two persons may have the same absolute SMM but different relative (%) amounts of SMM (percent skeletal muscle mass (PSMM)). However, the fact that two persons can also have different PSMMs because one person is fattier does not necessarily mean that the quality of their SMM (active movement potential) is different. This rather depends on the amount of protein mass that forms the muscles and amount of SMM per square unit of body size (i.e., BH [m^2^]). Therefore, this study aimed to explore the set of measures related to SMM in both sexes, and to create an age-related model of muscularity that is sex-sensitive. Furthermore, it aimed to determine the most representative indicator of muscularity obtained by InBody BIA machine.

## 2. Materials and Methods

A multicentric transversal survey study was applied in this study, while measurements were conducted in laboratory setting. Concerning the form, this study had characteristics of fundamental and applied research. It provided new insights in the area of understanding of changes in SMM characteristics through the adulthood and ageing of a general Serbian population of both sexes. The subject sample was gathered via a randomized method with a combined approach to the selection of respondents. The sample was gathered in the following ways: as part of regular screening, through measurement announcements given through the media, through personal acquaintances and the systematic testing of different companies, and testing of educational, medical and police personnel. The Institutional Ethical Board of Faculty of Sport and Physical Education, University of Belgrade Serbia, approved the study (No. 484-2). The research was conducted following the Helsinki Declaration: Recommendations Guiding Physicians in Biomedical Research Involving Human Subjects [35]. 

### 2.1. Subject Sample

The sample in this research consisted of 8733 subjects, among which 3370 (38.6%) were females (age = 33.8 ± 9.9 years; BH = 168.7 ± 7.4 cm; BM = 67.4 ± 13.2 kg; BMI = 23.74 ± 4.81 kg/m^2^), and 5363 (61.4%) males (age = 34.8 ± 9.1 years; BH = 182.2 ± 7.3 cm; BM = 87.3 ± 14.5 kg; BMI = 26.28 ± 3.98 kg/m^2^). Subjects were divided into six subsamples according to age, following the recommendations of the World Health Organization (WHO): 18–29.9 (*n* = 4475, 51.2%); 30–39.9 (*n* = 2369, 27.1%); 40–49.9 (*n* = 1192, 13.7%); 50–59.9 (*n* = 410, 4.7%); 60–69.9 (*n* = 173, 2.0%); and 70.0–79.9 (*n* = 114, 1.3%) years. The subjects were residents of 61 cities with the following percentage representation in the regions: Belgrade and its suburbs 34.65%, Vojvodina 13.39%, Central Serbia 23.62%, Southern and Eastern Serbia 13.39% and Western Serbia 13.39%. Considering the amount of population in the Republic of Serbia from 18.0 to 79.9 years, it could be stated that the study sample represents 0.17% of the overall national population (0.21% of males and 0.13% of females) [36]. All subjects were informed about the purpose of the study and only those who willingly consented to it were assessed.

### 2.2. Body Composition Measurement Procedure

All measurements were realized in the period from 2015 to 2019 in the Methodical Research Laboratory of the Faculty of Sport and Physical Education, University of Belgrade, in the Research Laboratory of the Faculty of Sport and Physical Education, University of Nis, as well as at the Institute of Hygiene and Medical Ecology of the Medical Faculty, University of Belgrade. Body composition assessments were conducted using a direct segmental eight-channel bioimpedance analyzer InBody 720 (Biospace Co., Ltd., Seoul, Korea). InBody 720 was shown to be valid, reliable and sensitive elsewhere [8,15,22,37]. The same official InBody service provider (Borf d.o.o, Belgrade, Serbia) calibrated all machines twice a year (once each six months). All subjects were measured according to the suggestions of the device manufacturer and following all test recommendations [8,10,38]. All measurements were carried out before breakfast between 08:00 and 10:00 h by experienced examiners. 

### 2.3. Variables

The definition of the subjects’ body composition took into account four types of variables. Besides the primary body composition variables considering absolute amount of contractile response tissue such as SMM, PM, and Free Fat Mass (FFM), there were three types of index variables: voluminosity-independent, longitudinality-independent, and combined body tissue index variable. Thus, the subjects’ contractile tissue was described independently of body dimensions. In total, nine variables were included: 3 primary variables, 2 voluminosity-independent variables, 3 longitudinality-independent variables, and 1 combined body tissue index variables. This approach was previously used in studies on body composition [6,8,10,20,39,40].

#### 2.3.1. Primary Body Composition Variables

- FFM—fat-free body mass, expressed in kg;
- PM—total body protein mass, expressed in kg;
- SMM—skeletal muscle mass, expressed in kg;

#### 2.3.2. Longitudality-Independent Body Composition Variables

- FFMI—fat-free mass index, expressed in kg/m^2^;
- SMMI—body skeletal muscle mass index, expressed in kg/m^2^;
- PMI—body protein mass index, expressed in kg/m^2^.

#### 2.3.3. Voluminosity-Independent Body Composition Variables

- PSMM—percent skeletal muscle mass, expressed in %;
- PP—percent protein mass, expressed in %.

#### 2.3.4. Combined Body Tissue Index Variables

- PFI—protein and total body fat ratio index.

### 2.4. Statistics

All row data were analyzed using the descriptive statistical procedure to calculate the basic measures of central tendency and dispersion of data such as Mean and Standard Deviation (SD). Correlations between explored muscle mass indicators and age were established using Pearson’s correlation analysis. With the Fisher t-to-z transformation for an independent sample, we calculated if the correlations were significantly different between sexes. General differences according to sex concerning age and muscle mass variables were tested using multiple (MANOVA) and differences between analyzed age groups were established by applying an analysis of variance (ANOVA). Principal Component Analysis with direct Oblimin rotation and Kaiser normalization was used to reduce the set of morphological variables into factors. Thereafter, the most representative variable that is projected at the first position of the extracted factor was used as the best representative of muscle tissue obtained by the BIA used in this study for the next step of statistical analysis. Finally, the method of mathematical modeling was used for defining specific age-muscle equations to determine the dependence of the change in muscular status as function of age relative to sex. In this way, it was possible to determine the model characteristics of the change for most sensitive variable in a function of time and to define the biological peak muscular potential relative to other age periods. All statistical analyses were performed using corresponding statistical software IBM SPSS Statistics 23.0. The level of statistical significance was defined at 95% and the probability values of *p* < 0.05.

## 3. Results

All descriptive data are shown in the form of Mean ± SD relative to sex and age subgroups (Table 1). Significant differences occurred between the age groups in all variables in males (Wilks’ Lambda Value = 0.473, F = 55.81, *p* = 0.000, Partial Eta^2^ = 0.086 [8.6%], Observed Power, 1.000 [100%]) and females (Wilks’ Lambda Value = 0.714, F = 25.67, *p* = 0.000, Partial Eta^2^ = 0.065 [6.5%], Observed Power, 1.000 [100%]).

Table 2 presents sex-specific correlation coefficients between age and indicators of muscularity, and Fisher r-to-z transformation for differences in correlation between sexes. The age correlated significantly with PM, PP, SMM, PSMM, PFI, FFM, and FFMI in males, and with PP, PMI, PSMM, SMMI, PFI, and FFMI in females. However, correlations were significantly higher in males in PP, PSMM and PFI, and significantly higher in females in PMI, SMMI, and FFMI. 

Kaiser–Meyer–Olkin measure of sampling adequacy and Bartlett’s test of sphericity were statistically significant for males (KMO = 0.663, Bartlett’s test = 193,878.5, *p* < 0.001) and females (KMO = 0.628, Bartlett’s test = 109,144.7, *p* < 0.001), which implies statistically significant suitability of data for complex multidimensional statistical analysis. Principal component analysis extracted two main components for males and two for females (Table 3), cumulatively explaining 89.8 and 90.96%, respectively, of the variance in muscularity. The individual component loadings were 59.08 and 30.74% for males, and 57.81 and 33.15% for females. 

The structure matrix sorted the variables by size of the loading for each both factors in both sexes (Table 3). The highest loaded, thus the most discriminative variable within the first component of both sexes, was SMMI, while within the second component it was PSMM. These two variables had the highest degree of methodological sensitivity and explained the highest amount of the variance in the muscularity of males and females. Therefore, they entered the regression analysis to determine the age–muscle relationship for both sexes.

**Figure 1:**
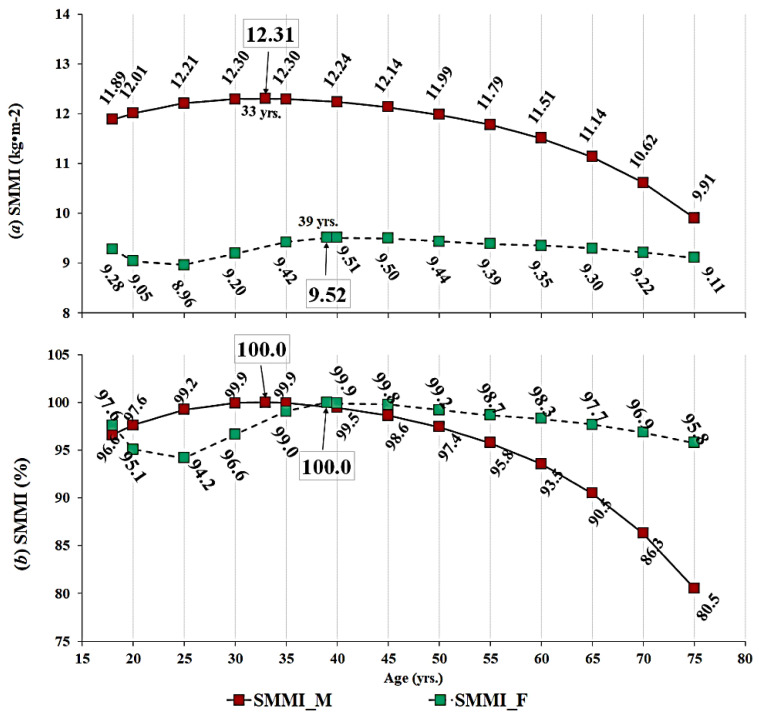
Gradually lower SMMI values across age groups presented in absolute (**a**) and normalized (**b**) values.

The regression analysis for age-predicted SMMI for males and females was conducted on absolute values (Figure 1a), which was followed by the SMMI normalized to maximal obtained SMMI (Figure 1b). The highest SMMI in males occurred at the age of 33 years and in females at the age of 39 years. Considering PSMM, the highest values occurred at the age of 18 years in both sexes and then the values were gradually lower following the age groups from younger towards older. In females, the values were linearly lower, while the sinusoid-shape trend of PSMM in males seems to be stable from 40–60 years (i.e., remains stable).

## 4. Discussion

The main findings of this study revealed significantly lower indicators of SMM across a gradually older sample of adults, whereby the most sensitive indicators of these trends were SMMI and PSMM, regardless of sex. Modeling and controlling changes in skeletal musculature over the years in the general population is essential not only for studying humans and health science but also as a source of general health data that could provide accurate and precise guidance as to whether something is right or wrong. This would be practical support of knowledge in aging process of general population that could be compared with the international data through the specific age range.

In general, the average protein mass and SMM of Serbian adult females are lower than in males. The results suggest that an average Serbian male possess about 50.69% higher SMM and 54.53% higher protein mass than the average Serbian female. Considering the same variables relative to longitudinal dimensions, the difference of 15.49 and 32.36% could be observed in PMI and SMMI. In relation to body voluminosity, males possessed about 15.40% higher PP, and 16.20% higher PSMM. This was further reflected in the 48.89% higher FFM and 27.54% higher FFMI of males. Although body fat mass was not analyzed, the PFI showed that the ratio of protein mass (i.e., contractile mass) to body fat mass was more than twice lower (108.1% lower) in females than in males, which clearly indicates higher adiposity in females. A dimorphism between sexes has been shown to exist elsewhere [3,41,42], while numerical determination of its size on a population level may have great implication in public health and physical fitness. Acceptable levels of body fatness have been thoroughly defined in the literature for both sexes, along with clearly defined differences [43,44], while the information on between-sex differences in muscularity is scarce. Considering that skeletal muscles are responsible for every movement that humans make, along with the movements’ strength, power, and precision, it is important to define the possible differences (highs and lows) within the humans of different sizes and certain biological characteristics (i.e., males and females). 

In that regard, the results showed significant difference between age groups in all investigated variables, indicating that higher initial (i.e., beginning of adulthood) amount of contractile tissue may be beneficial for ageing, as the gradual loss of skeletal muscles is likely to occur. The highest statistical difference occurred in PP, while the smallest was in FFM, meaning that the variable for the analysis of age-related, within-sex differences in muscularity should be chosen carefully for valid and meaningful evaluation (Table 1, Male PP, F = 411.46, PE^2^ = 0.28, FFM F = 9.69, PE^2^ = 0.01, Female PP, F = 156.64, PE^2^ = 0.19, FFM F = 10.55, PE^2^ = 0.02). This was additionally confirmed by the highest correlation between PP and age in males and females (see Table 2). PM is a building block of skeletal muscles and other cells, which directly depends on nutrition (or malnutrition) and adaptation to physical activity (or hypokinesia). Both nutrition and physical activity initiate the synthesis of proteins, whereby the type of nutrition and physical activity or exercise define weather PM will increase or remain the same [27,45,46]. However, because PM is an integral part of body composition and can remain the same even if the body composition changes (i.e., body fat increases), its relative (%) amount seems more valid for comparison between adults of different age. It is of note that the indicators of muscularity correlated to age differently in males and females, with larger variations in PP, PSMM, and PFI in males compared to females, suggesting that PP and PSMM decrease by age due to an increase in body fatness to larger extent than in females. In contrast, females seem to lose more pure contractile tissue, as PMI, SMMI and FFMI correlated more strongly in females than in males. Therefore, it seems that males are more prone to changes in ratio of fat and muscle tissue, mostly on account of body fat increase, while females, although more prone to loss of SMM, are affected by both. These variations are often reflected in physical performance when fat mass (ballast mass) increases over the criterion level, thus lowering PP and PSMM, when it becomes heavy enough to hinder the outcomes of muscle contractions, resulting in lower performance [3,20]. Given that the metabolism slows down by age, and an increase in body fatness is likely to occur, a good PP in adulthood, and its maintenance throughout aging could have a preventive effect on health and performance (hence quality of life), which could be obtained by regular physical activity and good eating habits.

This was additionally proved via principal component analysis that extracted SMMI and PSMM as the highest loaded variables in component 1 and 2, respectively, in males as well as in females (see Table 3). Therefore, longitudinally independent and body volume independent indicators of SMM are representatives of ageing in humans of different sizes within both sexes. SMM per each m^2^ of the body height controls for body size, so the SMM of smaller individuals can be compared to individuals of bigger body frame. In addition, PSMM is in direct opposite relation with percent of body fat, meaning that changes in PSMM are also reflected in percent of body fat, and vice versa. Considering this, these two variables entered the regression analysis revealed biological pick in SMMI and PSMM in males and females. The highest SMMI in males and females occurred at the age of 33 and 39 years, respectively. The lowest values in both variables were observed at the age of 75 years, with a difference of 19.5% in males and 4.2% in females compared to the pick values (see Figure 1). Compared to European consensus on sarcopenia definition and diagnosis [14], SMMI for 70 and more years old subjects corresponds to moderate sarcopenia (8.51–10.75 kg/m^2^) in males, while the SMMI of females corresponds to normal muscular status (5.76–6.75 kg/m^2^). Even though the regression R^2^ was not statistically significant, the trend of decrease in SMMI could be noticed. In contrast, non-significant regression indicate that not all subjects from gradually older groups had lower SMMI than the groups who were younger, providing evidence that raw muscular tissue could be maintained, or its loss could be slowed down through ageing. 

**Figure 2:**
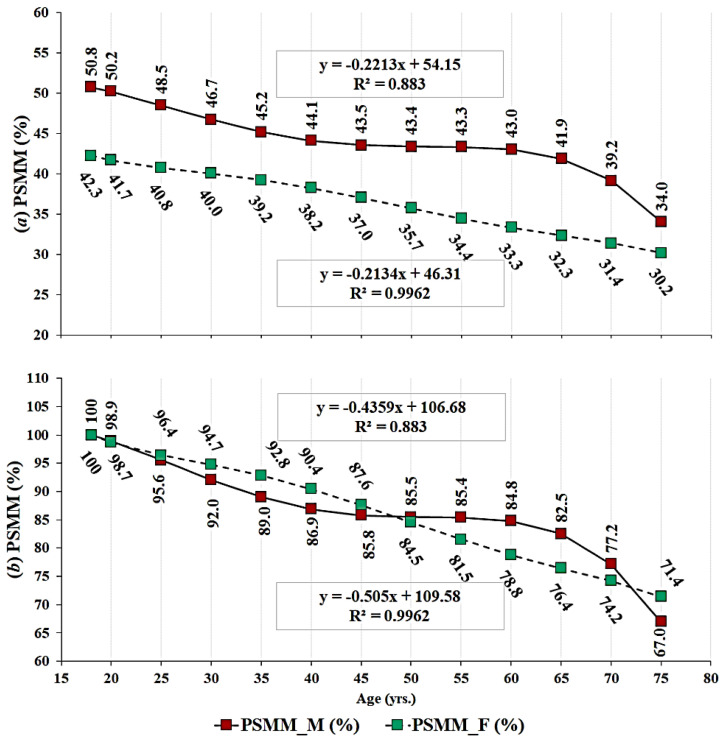
Gradually lower PSMM values across age groups presented in obtained (**a**) and normalized (**b**) values.

However, even if SMMI does not significantly change, the proportion of muscular tissue in the body can vary depending on the proportion of body fat. This could be observed from the regression analysis for PSMM that determined a strong negative, mostly linear relationship between age and PSMM in males and females (see Figure 2). The only exception seems to be in males between the ages of 45 and 55 years, when the PSMM is relatively stable and then continues to get lower. This can be qualitatively observed when analyzed relative to pick potential in PSMM that occurred at the age of 18 years, as PSMM at the ages of 45, 50 and 55 years was 85.5, 85.4, and 85.4% of the maximum, respectively. The difference between the pick (18 y) and lowest (79 y) relative muscular potential (PSMM) was 33% in males and 28.6% in females, whereby 99.6% and 88.3% of difference in PSMM could be explained by age in males and females, respectively. Based on the results, it could be stated that the PSMM in Serbian males and females decreases by a constant of −0.221 and −0.213% per each year of life between 18 and 79 years of age (see Figure 2a). Janssen et al. [22] reported lower constant values, −0.188 and −0.084%, for males and females. Our results showed that calculating from the values relative to pick value, the PSMM decreases about −0.505 and −0.436% each year, for male and female, respectively (see Figure 2b). These values were somewhat higher than those reported in review by Mitchell et al. [33], where median values were 0.47 for males and 0.37% for females. It is of note that results may vary depending on the method used, sample size and characteristics, cultural and socioeconomic effects on body composition [47,48]. However, although some differences exist between the studies in the size of differences in muscularity related to ageing, the trend of changes is the same and consensus exists on the significance of the effect of ageing on indicators of muscularity. 

The main limitations of this study could be the sizes of subsamples for subject groups 60–69.9 and 70–79, which could be larger. However, the obtained samples were still large enough to draw valid conclusions considering the aim of the study and the overall sample, and result in theoretical knowledge that already exists. Nutritional habits, physical activity levels and physical fitness measures may be beneficial to support an explanation of the obtained indicators of muscularity, even though the extensive literature is consistent in that regard, which we used to draw our conclusions. For higher accuracy, the DEXA method could be used as it would determine the most accurate reference values for Serbian population. However, this study used a more practical approach that is more likely to be employed in the public and private health sector as well as in the public and private sports sector. Therefore, that is how the results and conclusions from this study should be comprehended, which adds to strength of the practical application of the results.

## 5. Conclusions

Skeletal muscle mass is the biggest tissue in human body and responsible for all movements that we directly control in daily activities. During the growth phase of life, skeletal muscles are developing, building the potential for adulthood and ageing. However, results showed that all indicators of muscularity were gradually lower in older subjects. Because males and females differ in size of body frame within their sexes, indicators of skeletal muscles should be chosen to control for longitudinal differences. Furthermore, because body volume can vary significantly, even though the raw muscle tissue did not change, skeletal muscles should be evaluated as a proportion of total body dimensions. In that regard, SMMI and PSMM emerged as the most methodologically sensitive and precise indicators of muscularity in both sexes because they show the amount of muscles that a person has regardless of the size of their body frame, as well as the amount of skeletal muscles relative to other tissues in the body (i.e., body fat). This is of the utmost importance as even good SMMI is not necessarily sufficient for good health and performance if overloaded with overly increased body fat mass (i.e., ballast tissue). Conversely, sufficient PSMM does not necessarily mean good quality of skeletal muscles but merely a good proportion of muscles compared to body fats (i.e., indicating an underweight person due to malnutrition). Therefore, SMMI and PSMM both are important to follow during ageing because the results clearly showed a gradual decrease in both indicators, whereby the decrease was highly significant in PSMM. 

## Figures and Tables

**Table 1:** Descriptive data according to gender and age subgroups.

Sex	Variables	18–29.9	30–39.9	40–49.9	50–59.9	60–69.9	70–79.9	ANOVA
		Mean ± SD	Mean ± SD	Mean ± SD	Mean ± SD	Mean ± SD	Mean ± SD	
Males	PM (kg) **	14.16 ± 1.76	14.19 ± 1.81	14.06 ± 1.71	13.64 ± 1.65	13.01 ± 1.72	11.05 ± 1.59	F = 14.54, PE^2^ = 0.01
	PP (%) **	17.14 ± 1.36	15.65 ± 1.46	15.36 ± 1.34	15.02 ± 1.11	14.32 ± 1.52	13.58 ± 0.89	F = 411.46, PE^2^ = 0.28
	PMI (kg/m^2^) **	4.22 ± 0.35	4.28 ± 0.38	4.28 ± 0.37	4.2 ± 0.29	4.05 ± 0.41	3.72 ± 0.31	F = 14.59, PE^2^ = 0.01
	SMM (kg) **	40.73 ± 5.31	40.78 ± 5.53	40.4 ± 5.13	39.12 ± 4.98	37.24 ± 5.19	30.91 ± 4.82	F = 14.86, PE^2^ = 0.01
	PSMM (%) **	49.27 ± 3.96	44.95 ± 4.28	44.12 ± 3.88	43.04 ± 3.17	40.94 ± 4.3	37.94 ± 2.68	F = 409.57, PE^2^ = 0.28
	SMMI (kg/m^2^) **	12.13 ± 1.06	12.3 ± 1.18	12.29 ± 1.13	12.04 ± 0.9	11.59 ± 1.23	10.39 ± 1.03	F = 14.73, PE^2^ = 0.01
	PFI (kg) **	1.596 ± 0.98	0.874 ± 0.46	0.784 ± 0.40	0.673 ± 0.22	0.599 ± 0.23	0.466 ± 0.08	F = 273.55, PE^2^ = 0.20
	FFM (kg) **	71.1 ± 8.95	71.49 ± 9.18	71.00 ± 8.86	69.01 ± 8.39	66.91 ± 9.43	57.13 ± 8.63	F = 9.69, PE^2^ = 0.01
	FFMI (kg/m^2^) **	21.19 ± 1.75	21.56 ± 1.90	21.6 ± 1.94	21.24 ± 1.46	20.84 ± 2.37	19.20 ± 1.76	F = 14.60, PE^2^ = 0.01
Females	PM (kg) **	9.31 ± 1.37	9.55 ± 1.09	9.49 ± 1.04	9.02 ± 1.11	9.19 ± 1.54	7.89 ± 0.67	F = 11.54, PE^2^ = 0.02
	PP (%)	14.76 ± 1.56	13.95 ± 1.79	13.43 ± 1.61	12.54 ± 1.51	11.86 ± 1.48	11.84 ± 1.09	F = 156.64, PE^2^ = 0.19
	PMI (kg/m^2^) **	3.22 ± 0.31	3.34 ± 0.33	3.37 ± 0.29	3.33 ± 0.32	3.45 ± 0.48	3.29 ± 0.17	F = 30.30, PE^2^ = 0.04
	SMM (kg)	26.09 ± 4.17	26.81 ± 3.31	26.66 ± 3.13	25.22 ± 3.34	25.73 ± 4.66	21.75 ± 2.05	F = 11.61, PE^2^ = 0.02
	PSMM (%) **	41.29 ± 4.49	39.14 ± 5.04	37.66 ± 4.55	35.01 ± 4.21	33.13 ± 4.09	32.63 ± 3.07	F = 151.77, PE^2^ = 0.19
	SMMI (kg/m^2^) **	9.03 ± 0.98	9.37 ± 1.01	9.46 ± 0.87	9.29 ± 0.96	9.67 ± 0.46	9.09 ± 0.54	F = 26.12, PE^2^ = 0.04
	PFI (kg) **	0.671 ± 0.31	0.563 ± 0.28	0.473 ± 0.20	0.378 ± 0.14	0.332 ± 0.15	0.322 ± 0.07	F = 92.77, PE^2^ = 0.12
	FFM (kg) **	47.34 ± 6.87	48.67 ± 5.51	48.43 ± 5.33	46.18 ± 5.82	47.49 ± 8.25	41.53 ± 4.30	F = 10.55, PE^2^ = 0.02
	FFMI (kg/m^2^) **	16.39 ± 1.53	17.02 ± 1.63	17.19 ± 1.46	17.03 ± 1.66	17.86 ± 2.75	17.34 ± 1.07	F = 40.34, PE^2^ = 0.06

** Significant at *p* < 0.01. PM—protein mass, PP—percent protein, PMI—protein mass index, SMM—skeletal muscle mass, PSMM—percent of SMM, SMMI—skeletal muscle mass index, PFI—protein/fat index, FFM—fat-free mass, FFMI—fat-free mass index, PE^2^—Partial eta square.

**Table 2:** Pearson correlation results between age, basic anthropometrics and other muscle mass variables with Fisher r-to-z transformation results.

Variables	Pearson Correlation		Fisher r-to-z Transformation	*p*
	Male	Female		
PM (kg)	−0.065 **	−0.034	−1.413	0.079
PP (%)	−0.517 **	−0.443 **	−4.378	0.000
PMI (kg/m^2^)	0.015	0.165 **	−6.890	0.000
SMM (kg)	−0.066 **	−0.033	−1.504	0.066
PSMM (%)	−0.516 **	−0.438 **	−4.598	0.000
SMMI (kg/m^2^)	0.008	0.148 **	−6.416	0.000
PFI (kg)	−0.432 **	−0.354 **	−4.199	0.000
FFM (kg)	−0.038 *	−0.010	−1.274	0.101
FFMI (kg/m^2^)	0.055 **	0.200 **	−6.716	0.006

* Significant at *p* < 0.05, ** Significant at *p* < 0.01.

**Table 3:** Structure matrix extracted according to the gender.

Variables	Component—Female		Variables	Component—Male	
	1	2		1	2
SMMI	0.939	−0.135	SMMI	0.946	−0.040
SMM	0.935	0.196	SMM	0.944	0.151
PM	0.934	0.200	PM	0.943	0.147
FFM	0.928	0.175	FFMI	0.938	−0.102
PMI	0.926	−0.163	FFM	0.937	0.116
FFMI	0.919	−0.201	PMI	0.934	−0.059
PSMM	0.015	0.983	PSMM	0.041	0.977
PP	−0.062	0.980	PP	−0.018	0.975
PFI	0.078	0.930	PFI	0.079	0.897
